# Retrospective analysis of incidental non-trauma associated findings in severely injured patients identified by whole-body spiral CT scans

**DOI:** 10.1186/s13037-014-0036-3

**Published:** 2014-08-31

**Authors:** Johannes KM Fakler, Orkun Özkurtul, Christoph Josten

**Affiliations:** 1 Department of Orthopaedic and Orthopaedic Trauma Surgery, Reconstructive Surgery, University Hospital of Leipzig AöR, Liebigstr. 20, Leipzig 04105, Germany

**Keywords:** Whole-body CT, Multiple injured, Polytrauma, Incidental findings

## Abstract

**Background:**

Whole-body Computed Tomography (CT) scan today is considered a crucial imaging technique in the diagnostic work-up of polytrauma patients implicating a potential survival benefit. Apart from prompt identification of life threatening injuries this imaging technique provides an additional benefit by diagnosing incidental non-trauma associated medical diseases. These incidental findings might be also life threatening and warrant urgent therapy. The downside of whole-body CT is a relatively high radiation exposure that might result in an increased life time cancer risk. The aim of this study was to investigate the frequency and type of non trauma associated incidental medical findings in relation to patient age and potential clinical relevance.

**Methods:**

Between January 1^st^ 2011 and December 15th 2012, a total of 704 trauma patients were referred to our hospital’s emergency room that triggered trauma room alarm according to our trauma mechanism criteria. Of these 534 (75.8%) received a whole-body CT according to our dedicated multiple trauma protocol. Incidental Findings (IF) were assigned in three groups according to their clinical relevance. Category 1: IF with high medical relevance (urgent life threatening conditions, unless treated) needing early investigations and intervention prior to or shortly after hospital discharge. Category 2: IF with intermediate or low medical relevance, warranting further investigations. Category 3: IF without clinical relevance.

**Results:**

Overall 231 IFs (43.3%) were identified, 36 (6.7%) patients had IFs with a high clinical relevance, 48 (9.0%) with a moderate or minor clinical relevance and 147 (27.5%) with no clinical relevance. The distribution of incidental findings with high or moderate relevance according to age showed an incidence of 2.6%, 6.6% and 8.8% for patients younger than 40 years, 40 to 60 years and older than 60 years, respectively.

**Conclusion:**

Whole-body CT scans of trauma patients demonstrate a high rate of incidental findings. Potentially life-threatening, medical findings were found in approximately every 15th patient, predominantly aged over 40 years and presenting with minor to moderate injuries and an Injury Severity Score (ISS) of 10 or less.

## Background

Whole-body Computed Tomography (CT) scan today is considered a crucial imaging technique in the diagnostic work-up of polytrauma patients [1]-[3]. The multidetector approach in particular provides appropriate diagnostic algorithms for detecting relevant trauma findings with a high grade of sensitivity and specificity. This can be explained by recent technical improvements in CT scanner hard- and software delivering high-quality images of various body parts within a short time [4]-[6]. While several studies so far have demonstrated the technical feasibility of whole-body CT introducing various protocols and contrast media application techniques [7],[8], the exact role and clinical value of a whole-body CT in terms of non-trauma-associated additional findings, as compared to potential estimated radiation risks, is still a matter of debate. A recent multi-center trial suggested that the integration of whole-body CT into early trauma care significantly increases the probability of survival in polytrauma patients when compared to those undergoing non-whole-body CT [1]. The downside of whole-body CT is the relatively high radiation exposure, which has led to further debates about the value of this approach especially in younger patients or elderly patients with minor injuries [9]. Although the clinical significance of incidental findings on whole-body-CT has been reported, the success of trauma physicians in managing these findings, especially under consideration of mild trauma in elderly patients, has not been studied yet [10],[11]. The radiation issue is pertinent because CT examinations result in much higher organ doses than those with conventional single-film x-rays [12]. The radiation dose from whole-body CT is 10–20 mSv, which results in an estimated lifetime cancer mortality for 45-year-old people of about one in 1250 or 0.08% [13]. There is also concern that CT is replacing physical examination, and a previous study [14] with even more liberal criteria than in today’s report was heavily criticised for unnecessary radiation exposure [15]. The indiscriminate use of CT for patients with minor injuries is not justified. The cohort reported by Huber-Wagner did not seem to be minimally injured. The aim of our study was to investigate the usefulness of whole-body CT for trauma assessment under consideration of non-trauma-associated age related medically relevant incidental findings and its clinical consequences.

## Methods

All patients included in this study provided written informed consent for participation. The Ethical Review Board of the University of Leipzig does not require a formal IRB review for retrospective studies, and the IRB approval was therefore waived for this study. Between January 1^st^ 2011 and December 15th 2012, a total of 704 trauma patients were referred to our hospital’s emergency room that triggered trauma room alarm according to our list of trauma room activation criteria:

 systolic blood pressure below 90 mmHg after trauma

 penetrating injuries to the neck and torso regions

 gunshot wounds to the neck and torso regions

 GCS below 9 after trauma

 respiratory impairment/requirement for intubation after trauma

 Fracture of more than 2 proximal bones

 Unstable chest

 Pelvic fractures

 Amputation injury proximal to hands/feet

 Spinal cord injury

 Open head wounds

 Burns > 20% and degree ≥ 2b

 fall from more than 3 meters height

 road traffic accident with

 frontal collision with intrusion by more than 50–75 cm

 a change in speed of delta > 30 km/h

 collision involving a pedestrian or two-wheeler

 death of a driver or passenger

 ejection of a driver or passenger

After initial management and evaluation according to the ATLS algorithm, 534 (75.8%) received a whole-body CT according to our dedicated multiple trauma protocol. The indication for a CT scan is given by the trauma leader according to the primary clinical assessment and the trauma impact. Additionally, the indication for a whole-body CT was verified by a radiologist, since no specific protocol in terms of indications for a whole-body CT exists. The whole-body CT examination protocol starts with a head scan. After injection of intravenous contrast media it is completed by scans of the neck, thorax, abdomen and pelvis. A 64-row multidetector CT was used in all patients (Brilliance, Philips Medical Systems, Cleveland, USA). The CT scans were analysed by a trauma surgeon and a radiologist in the acute setting for injuries. Thereafter the radiologists analysed the CT scans in detail and wrote the report. The definitive reports were reviewed by a trauma-surgeon for Incidental Findings (IF). Correspondent to relevance IFs were subdivided into the level of concern for serious pathology they generated in the mind of the physician reading the report. Because of the expected wide variety of potential findings, three deliberately general categories were chosen according to their clinical relevance. Category 1: IF with high medical relevance with mandatory further diagnostic work-up and potential intervention prior to or shortly after hospital discharge. Examples for this category are lesions highly suspicious of malignant disease, non-trauma-associated aortic aneurysms with a diameter more than five centimeters, high-grade stenosis of major arterial vessels (>80%), pneumonia, etc. Category 2: IF with intermediate or low medical relevance. Additional diagnostics are strongly recommended, but can be done after discharge in an out-patient setting. Examples are most likely benign lesions, aortic aneurysms with a diameter less than five centimeters. Category 3: IF without clinical relevance. Follow-up examinations or interventions are not necessary as for benign cysts of the kidney or liver. Sinusitis, mucus retention cysts, degenerative disease of the joints or vertebral column, age-related cerebral atrophy and hernias (except incarcerating hernias) were excluded.

Patients grouped into Category 1 had highly relevant findings and were considered clinically significant with acute or subacute life threatening situations. For these patients additional follow-up research was performed including Injury Severity Score (ISS), body region of incidentaloma and age related comorbidities. Additionally, CT reports were compared to discharge reports to verify that these findings were addressed correctly. To evaluate follow-up of the findings, hospital charts and computerized records were then reviewed to confirm that the findings were new, and to see if any follow-up investigations (histological analyses, laboratory testing, reimaging, or procedures) were subsequently performed.

Statistical analysis: Data are presented as mean values with Standard Deviation (SD±) in parentheses. A Student-t or Mann–Whitney statistical test was used to compare continuous normally or not normally distributed variables, while a Chi-Square or Fisher’ Exact test was used for categorical variables. Statistical analysis was performed with SPSS 20 (SPSS, Chicago, USA).

## Results

**Figure 1:**
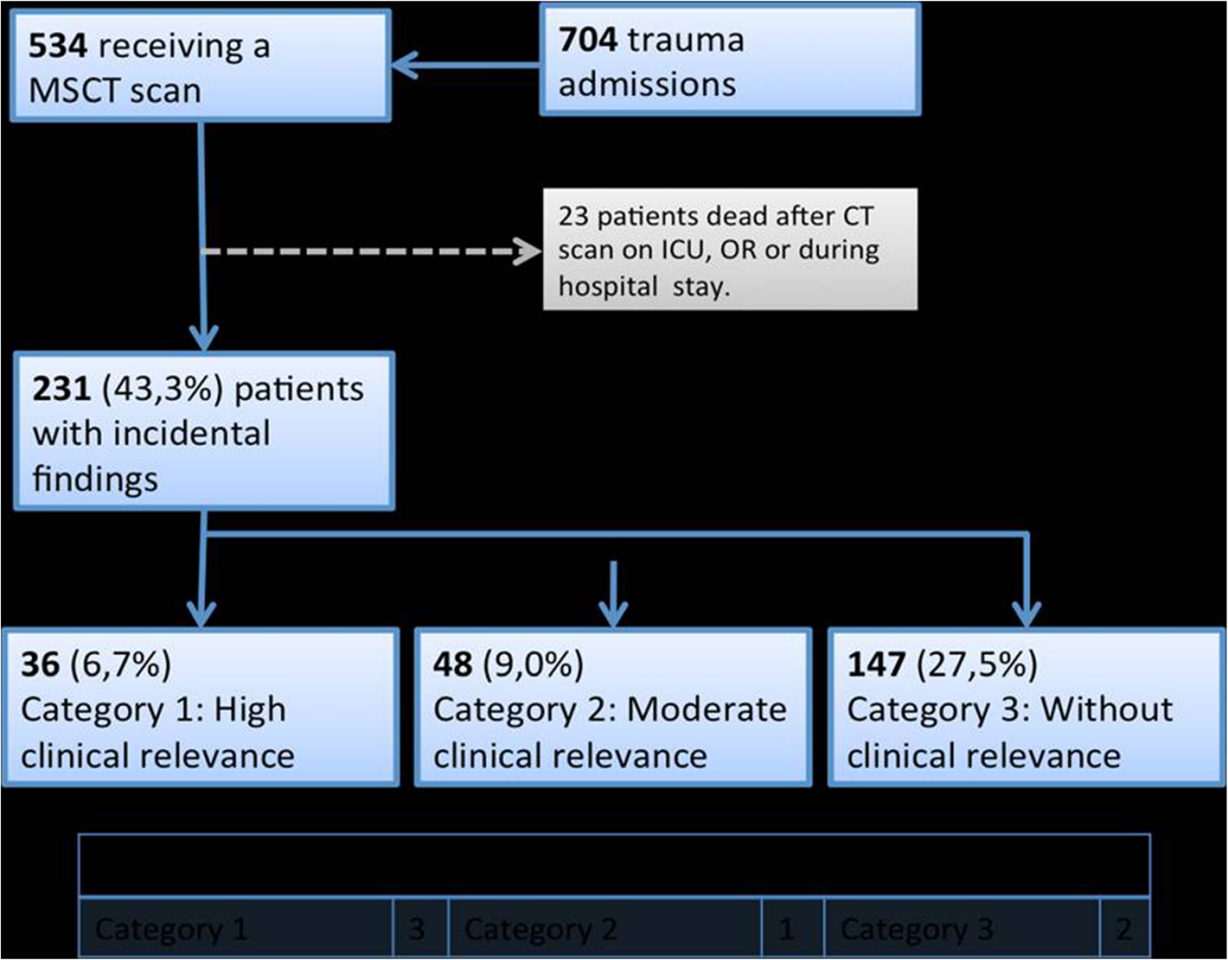
Flowchart demonstrating selection of study population.

**Figure 2:**
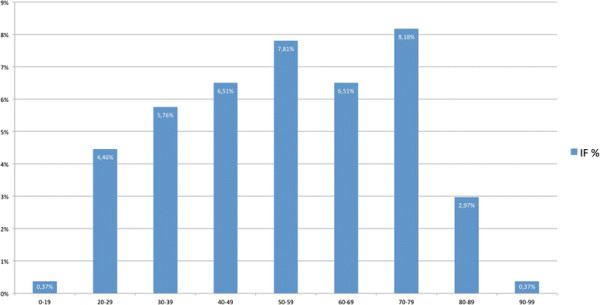
Identification of incidental findings (IF) increases with age, a decline can be noticed beyond 80 years.

**Figure 3:**
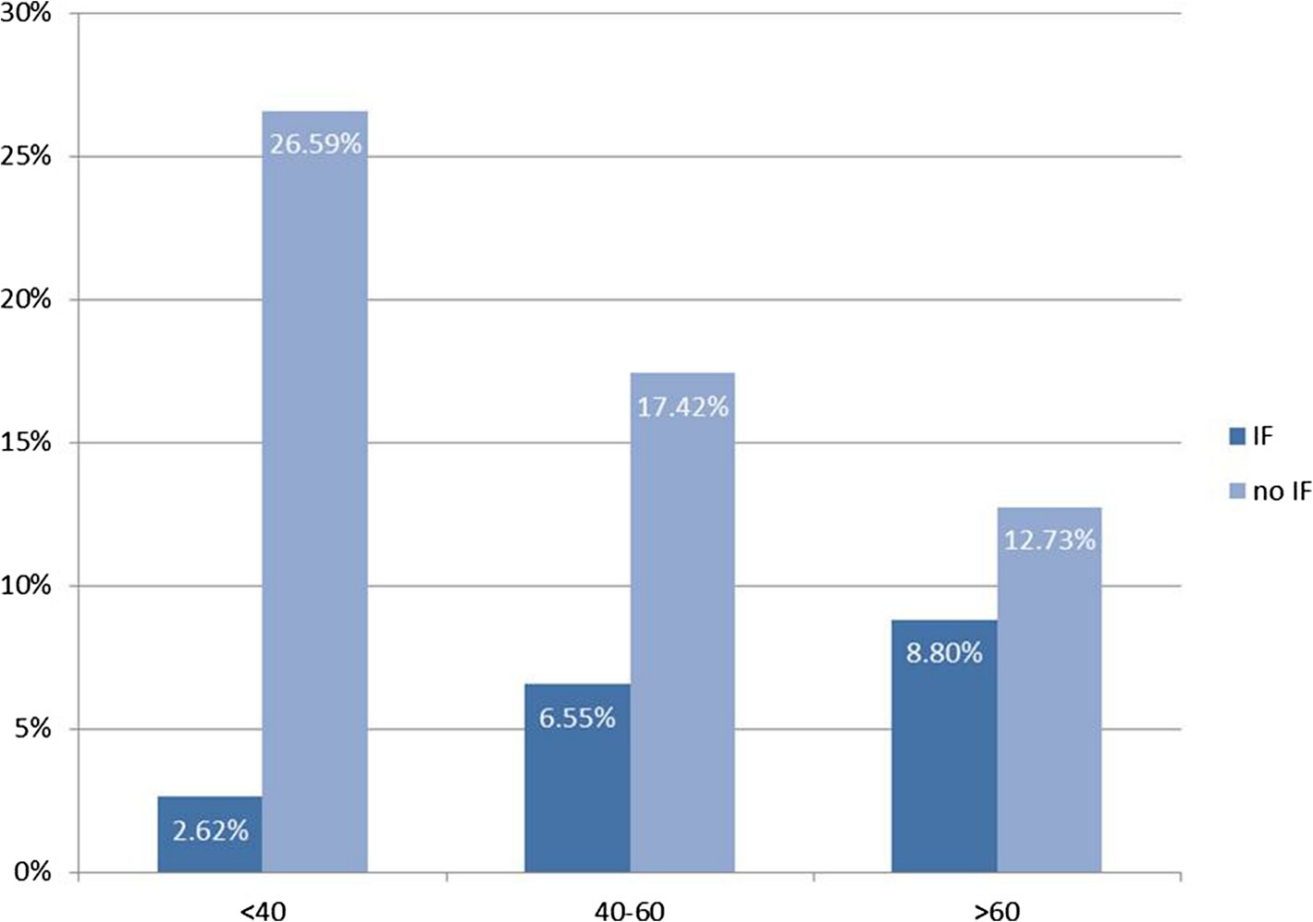
Incidental findings (IF) of moderate and high relevance are highest in the age group over 60 years.

**Figure 4:**
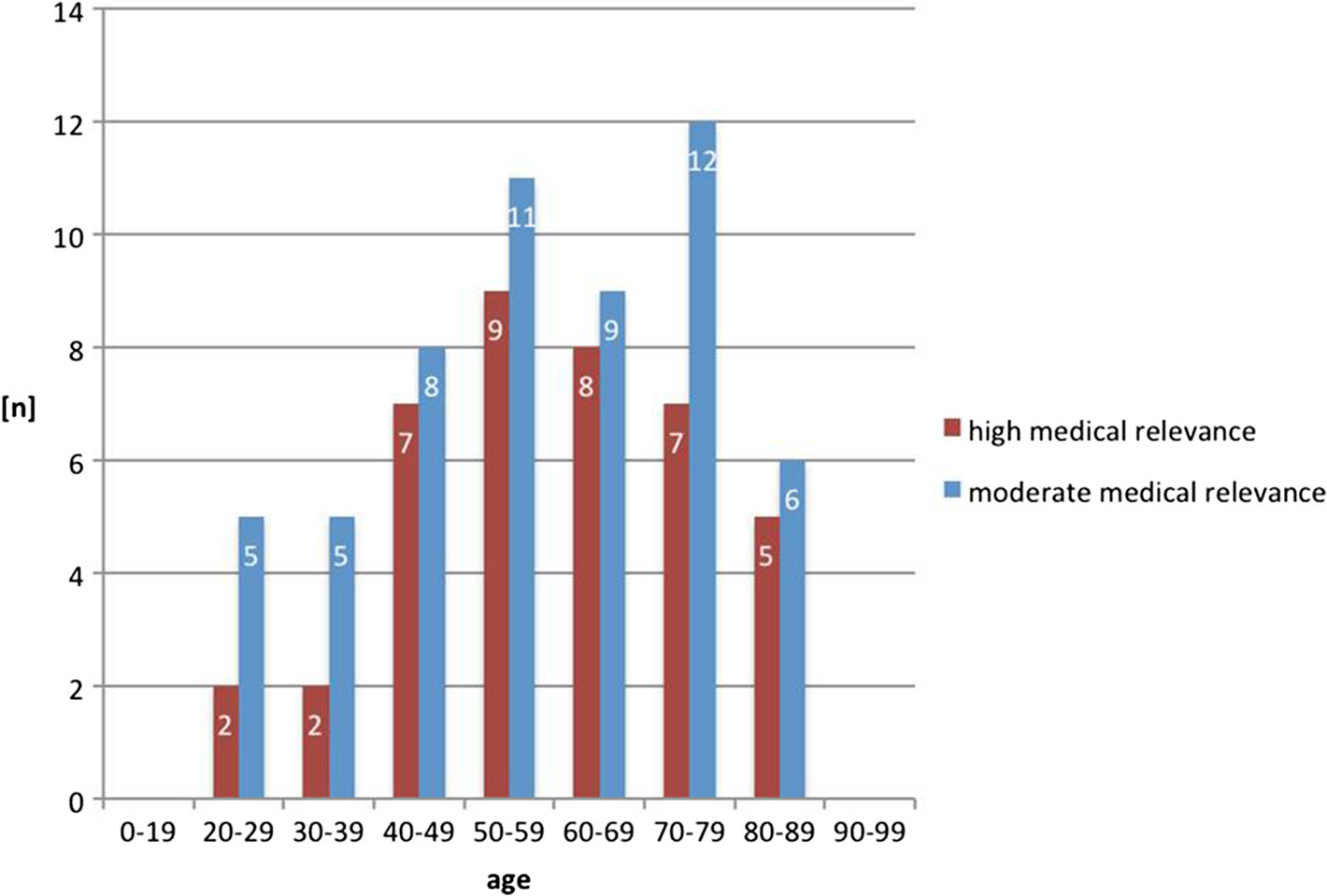
Incidental findings (IF) of high relevance peak in the age group 50–59 years, moderately relevant IFs show a double peak (50–59 years and 70–79 years).

534 patients were admitted over the study period and received whole body polytrauma CT as part of our trauma assessment. The mean age of the patients was 48 years (SD +/− 19.9 years, range 16–92). Gender distribution showed 377 (71%) male and 157 (29%) female patients (Table 1). 231 (43.3%) patients were identified as having an IF. 155 (67.1%) of these patients were male, 76 (32.9%) female. A breakdown of patient acquisition is shown in Figure 1, with 231 eligible patients included in the study population. Overall, 36 (6.7%) patients had incidental findings with a high clinical relevance, 48 (9.0%) with a moderate or minor clinical relevance and 147 (27.5%) with no clinical relevance. Interestingly all 16 patients with an ISS ≤10 (3.0%) demonstrated Category 1 incidental findings (Table 2). IFs were located in the head in 30 (11.2%) patients, 16 (6.1%) in the neck, 54 (20.7%) in the thorax, 129 (49.4%) in the abdomen and 32 (12.3%) in the pelvis area. In 37 cases (6.9%) more than one IF in a patient could be located in other body regions so there was a total of 497 IFs. The age distribution of patients with IFs of all categories is shown in Figure 2. The distribution of all incidental findings with respect to the three age groups, younger than 40 years, 40–60 years and older than 60 years was 10.7%, 14.4% and 18.2% (Figure 3). Referring only to IFs with moderate or high relevance a more pronounced increase was observed with age (2.6%, 6.6%, 8.8%, p > 0.05). When comparing patients younger than 40 years with those aged 40 or older, the difference becomes even more striking with a probability of moderately to highly relevant IFs being more than three times higher in the latter group. Absolute numbers of moderately and highly relevant IFs according to age decades can be obtained from Figure 4. For 138 (26%) of the 534 patients receiving whole-body CT an ISS less than 16 was calculated and consequently regarded as overtriaged. Nevertheless, 12% of these showed highly relevant medical Ifs.

**Table 1:** Characteristics of study population

**Characteristics of study population:**		**534**	
Males	70,6% (377)		
Age (years)	48 ± 19,78	(Range 16–92)	
	<40 (n = 199)	40-60 (n = 170)	>60 (n = 165)
no IF	142 (26,6%)	93 (17,4%)	68 (12,7%)
with IF	57 (10,7%)	77 (14,4%)	97 (18,2%)

Incidental findings (IF) depending on age groups.

**Table 2:** Patients with category I incidental findings and ISS ≤10

**No.**	**Age**	**Gender**	**ISS**	**Reported finding**	**Trauma?**	**FU?**
106	57	♂	6	Duodenal ulcer	GSI	Yes
238	46	♀	1	Leriche-Syndrome, adrenal adenomas	MVA	No
258	83	♀	1	Suspected malignant lesion in the liver and lung	Fall <3 m	Yes
384	57	♂	1	Suspected malignant lesion in the lung, Enchondroma of the Humerus	Fall <3 m	No
391	71	♀	4	Suspected malignant lesion in the kidney	Fall <3 m	Yes
420	67	♀	8	Suspected malignant lesion in the kidney	MVA	No
452	21	♀	10	Hepatomegaly with suspected malignoma	Fall >3 m	No
464	38	♂	8	Liquefying necrotizing pneumonia	Fall >3 m	Yes
468	54	♂	6	Suspected pleural malignacy	Fall <3 m	No
469	24	♀	1	Suspected malignant lesion in the liver, ureterolithiasis	Acerbity accident	Yes
474	69	♀	1	Renal cell cancer	Fall < 3 m	Yes
492	47	♂	1	Suspected lung cancer	MVA	No
523	49	♀	4	Suspected liver cell cancer	MVA	No
527	54	♀	8	Metastatic ovarian cancer	MVA	No
695	69	♀	9	Renal cell cancer	Fall <3 m	No
696	69	♀	4	Suspicious complex uterus cyst, complex renal cyst, abdominal wall hernia	Fall <3 m	No

Patients with minor or moderate Injury Severity Score (ISS ≤ 10) and non-trauma associated incidental findings of high relevance (category I). MVA: Motor Vehicle Accident, GSI: Gun Shot Injury, FU: Follow-Up.

(♀)=female, (**♂**)=male.

A detailed review of the charts of patients with Category 1 incidental findings revealed consistently poor rate of documentation of both the incidental findings and the management or referral for these lesions (Table 3). Only 19 (52.8%) of 36 applicable Category 1 findings were mentioned in the discharge summaries. In only 10 (27.8%) cases additional diagnostics, interventions or referral to a correspondent medical specialist was documented. Documentation was missing or inadequate in 47.2% of all Category 1 discharge summaries.

**Table 3:** Patients with verification of incidental findings

**Pat.**	**Age**	**TAD**	**ISS**	**IF**	**DL**	**FU**	**Verification**
**99**	80	Pertrochanteric femoral fracture, floating shoulder, lung contusion	>16	Suspected Bladder carcinoma	Yes	Yes	Muscle invasive bladder carcinoma, at least pT2a, G2
**325**	71	Cervical spine fracture C6 with complete paraplegia	>16	Suspected Renal cell carcinoma	No	No	Nephrectomy 09/2012: T1A pNx M0 L0 V0 PNO R0 G2;
**391**	71	Abdominal bleeding	4	2 suspicious lesions on upper kidney pole	Yes	Yes	Nephrectomy 07/2012: pT3a(2), pNX, M0, L0, V0, Pn0, UICC-Stad. III, R0
**392**	34	TBI, Lumbar spine fracture L1, open lower leg fracture, calcaneal fracture, distal radial fracture	>16	Aortic aneurysm > 5 cm in diameter	Yes	Yes	Stent implantation, Class. Stanford B
**464**	38	Transverse process fracture of L1, TBI	8	Suspected melted pneumonia, suspicious shading of the lung	Yes	Yes	COPD with acute exacerbation
**505**	47	Acetabular fracture	>16	Suspected exsudative tuberculosis	Yes	Yes	Lung TBC, pathologic changes of lung tissue into squamous cell metaplasia

Patients with intraoperatively or histopathologically verified diagnosis of the suspected lesion in the CT-scan. TAD: Trauma associated diagnosis, ISS: Injury severity Score, IF: Incidental finding, DL: mentioned in Discharge letter, TBI: Traumatic brain injury, FU: Follow-up.

## Discussion

Consistent with our data incidental findings in whole body CT scans for severely injured patients are very prevalent with up to 50% [16],[17]. We detected incidental findings in 43.3% of 534 scans with 9.0% and 6.7% classified as moderately and highly relevant in terms of clinical importance, respectively. The majority of Category 1 findings were discovered predominantly on CT scans of the abdomen (49.4%) and the chest (20.7%). Similar results were obtained by others analysing single CT scans of the chest or other body regions [10],[11] Analogous to our results Hofstetter et al. [17] also found 6.6% incidental findings of clinically high importance in their series of 304 multiple injured patients receiving whole-body CT with a consistent protocol. Incidental findings of moderate importance were described in 22.7%, which is more than twice as high compared to our study cohort. This can be explained by different definitions of findings with moderate importance. Degenerative skeletal disease represented the largest portion of findings with moderate importance in the study of Hofstetter et al. [17]. Contrary, we excluded degenerative skeletal disease in our study population for two reasons. First, these diseases are very frequent, especially if mild forms are included. This potentially leads to an inappropriate positive interpretation of results. Second, degenerative disease usually has no clinical near- or midterm relevance. If at all, these diseases should be regarded as less important in our view. With a similar definition of Category 2 findings or findings of moderate clinical importance our results compare well to those of Paluska et al. [16]. On the other hand their rate of findings with high relevance was lower by approximately half with 3.1%. Since they didn’t use a consistent whole-body CT protocol, but applied region specific spiral CT-Scans to areas of suspected injuries, many incidental findings might have been missed.

We found clinically highly important IFs in 12% of overtriaged patients with comparatively minor to moderate injuries and an ISS < 16. The American College of Surgeons Committee on Trauma estimates that an overtriage rate of 30-50% is necessary in order to carry out efficient emergency room care [18]. Kane et al. demonstrated that in order to identify severely injured patients with a sensitivity of more than 80%, the rate of overtriage could not be brought below 70% [19]. Overtriage referring to whole-body CT in trauma patients is supposed to be as high as 30-66% [2],[20],[21]. On the other hand, implementation of whole-body CT has shown to reduce mortality in trauma patients [1]. Nevertheless, use of whole-body CT in the assessment of trauma patients is under debate due to relatively high radiation exposure and potential indiscriminate use [22]. The radiation dose of whole-body CT is estimated to be 10-20 mSv which results in an estimated lifetime cancer mortality for 45-year-old patient of about one in 1250 or 0.08% [13]. In our view, the overall risk of radiation exposure is outweighed by the chance of identifying possibly life threatening non-trauma associated clinical evidence in 12% of overtriaged trauma patients. Nevertheless, the trauma leader should be urged to verify the indication for whole-body CT properly, especially in patients younger than 40 years. On the other hand our study implies that whole-body CT might be used more liberally in elderly trauma patients. 40% of IFs with a moderate to severe clinical impact were found in the age group beyond 60 years in which life time cancer risks due to radiation presumably is less eminent [13]. Besides, age and comorbid conditions are independent risk factor for mortality in elderly trauma patients [23]. Additionally, minor trauma increasingly results in severe injuries among the older population [24]. Furthermore, morbidity due to complications as well as mortality is more than twice those seen in younger adult trauma patients [24],[25] underpinning a more liberal use of whole-body CT in the elderly.

The second key finding of this study was a poor work-up of IFs with high clinical relevance. 47.2% of these findings were not documented in the discharge reports. It might be hypothesized that a growing number of elderly trauma patients [9] with a high rate of concomitant comorbidities [26] increases the probability of additional unknown serious diseases as demonstrated by the high rate of major IFs in elderly patients in our study. Consequently, a potential delay in appropriate care and subsequent increased morbidity or even mortality might arise from not adequately addressed and documented IFs. Apart from ethical and socioeconomic issues, medicolegal implications must be considered in this context. Recent studies provide a potential organized approach or management pathway that can help address these difficult incidental radiographic issues [27]. The Incidental Finding Coordinator (IFC) documented incidental findings daily from trauma admission, improved notification and documentation of IFs and promoted appropriate follow-up of these patients. The implementation of an IFC resulted in more than a 2.5-fold higher capture of incidental findings. It also resulted in an almost complete initiation of patient follow-up and hospital record documentation of incidental finding events [27].

Several limitations to this study deserve mention. It was conducted at a single level 1 trauma-center and patients may have had follow-up at another institution. Only 50% of Category 1 findings have been verified in this study. Differences between trauma centers and detailed protocols used for diagnostic work-up and whole-body CT scans were not described. Accordingly, generalization of our results might be limited due to differences in management of trauma patients, quality of trauma care providers, and characteristics of the study sample. Regarding the diagnostic workflow, it was assumed that the radiologist reported all significant IFs. Minor findings as for degenerative skeletal diseases were not reported, subsequently our overall rate of IFs may be falsely low. Finally, no formal or verified classification of IFs is available. Therefore, a bias in determining the severity or extent of clinical impact with respect to IFs cannot be excluded.

## Conclusion

Whole-body CT scans of trauma patients demonstrate a high rate of incidental findings. An incidental finding of high medical importance, potentially life-threatening, was found in approximately every 15th patient. Moreover, 9% of these patients revealed IFs of minor to moderate medical relevance warranting further diagnostic work-up or treatment in the short- or mid-term. The diagnostic value of whole-body CT examinations are emphasized in elderly trauma patients aged 60 years or higher in which a more liberal use can be recommended in our view. Work-up and documentation of important IFs in discharge reports is poor and must be improved substantially in order to justify broader use of whole-body CT scans in elderly patients with minor or moderate trauma, as they have a higher rate of potentially life-threatening non-trauma associated new findings.

## Abbreviations

CT: Computed tomography

IF: Incidental finding

ISS: Injury severity score

SD: Standard deviation

IFC: Incidental finding coordinator

TAD: Trauma associated diagnosis

TBI: Traumatic brain injury

FU: Follow-up

MVA: Motor vehicle accident

GSI: Gun shot injury

## Competing interests

The authors declare that they have no conflicting interests.

## Authors’ contributions

JKMF conceived the study, analyzed and interpreted the data and drafted the manuscript. OÖ acquired data of the CT and discharge protocols and helped drafting the manuscript. CJ was involved in drafting the manuscript and revising it critically for important intellectual content and gave final approval of the version to be published. All authors read and approved the final manuscript. The study was conceived without funding.
